# UHPLC-ESI-QTOF-MS/MS Metabolite Profiling of the Antioxidant and Antidiabetic Activities of Red Cabbage and Broccoli Seeds and Sprouts

**DOI:** 10.3390/antiox10060852

**Published:** 2021-05-26

**Authors:** Simon-Okomo Aloo, Fred-Kwame Ofosu, Eric-Banan-Mwine Daliri, Deog-Hwan Oh

**Affiliations:** Department of Food Science and Biotechnology, College of Agriculture and Life Sciences, Kangwon National University, Chuncheon 24341, Gangwon-do, Korea; okomosimon@gmail.com (S.-O.A.); fkofosu17@gmail.com (F.-K.O.); ericdaliri@yahoo.com (E.-B.-M.D.)

**Keywords:** germination, antioxidant capacity, advanced glycation end products, amino acid, phenolic compounds

## Abstract

The antioxidant and antidiabetic properties and metabolite profiling of ethanol extracts of red cabbage (RC) and broccoli (BR) seeds and sprouts were investigated in this study. The total phenolic, flavonoid, and saponin contents were in the ranges of 385.4–480.4 mg FAE/100 g, 206.9–215.6 mg CE/100 g, and 17.8–27.0 mg soysaponin BE/100 g, respectively. BR seed had the highest total phenolic (480.4 mg FAE/100 g) and flavonoid (216.9 mg CE/100 g) contents, whereas BR sprout had the highest saponin content (27.0 soysaponin BE/100g). RC sprout demonstrated the highest antioxidant capacity, with DPPH and ABTS radical scavenging activity levels of 71.5% and 88.5%, respectively. Furthermore, BR and RC sprouts showed the most potent inhibition against α-glucosidase (91.32% and 93.11%, respectively) and pancreatic lipase (60.19% and 61.66%, respectively). BR seed (60.37%) demonstrated the lowest AGE inhibition. A total of 24 metabolites, predominantly amino acids and phenolic compounds, were characterized using UHPLC-QTOF-MS/MS. Germination not only improved the levels of metabolites but also resulted in the synthesis of new compounds. Therefore, these findings show that germination effectively enhanced the functional properties and metabolite profiles of broccoli and red cabbage seeds, making their sprouts more applicable as functional ingredients.

## 1. Introduction

Diabetes mellitus is one of the most serious and complex chronic illnesses causing deaths all over the world. It has been reported that the disorder affects about 4% of the world population and the prevalence is projected to surpass 5.4% by 2025 [1]. Diabetes is caused by a chain of complex reactions characterized by hyperglycemia and alterations in the metabolism of carbohydrates, protein, or lipids [2]. The chronic condition is associated with various acute metabolic side effects, such as ketoacidosis and hyperosmolar coma leading to chronic disorders, including retinopathy, renal failure, neuropathy, and other cardiovascular-related complications [3]. Moreover, elevated blood sugar may trigger non-enzymatic interactions between glucose and proteins, leading to the formation of glycated products known as advanced glycation end products (AGE) [4]. In diabetic patients, the accumulation of AGEs is accelerated, leading to further complications, including inflammation of tissues and formation of permanent crosslinks with body macromolecules [5]. Diabetes can be classified into insulin-dependent (IDDM) and non-insulin-dependent diabetes mellitus (NIDDM), with the latter being the most prevalent among diabetic patients [1]. The high prevalence rate of diabetes has led to various interventions, including developments of natural remedies and dietary management strategies steered toward finding safer alternatives to synthetic drugs in efforts to treat the disorder. From the perspective of dietary management, the development of antihyperglycemic foods that possess the potential to reduce the negative side effects of diabetes is a priority to researchers. Such foods can act as antioxidants, reducing inflammatory responses due to free radicles; as enzyme inhibitors blocking the hydrolyzing activities of α-glucosidase, amylase, and pancreatic lipase; and as AGE formation inhibitors preventing the glycation of fats or protein complexes in diabetic patients [1,4,5].

Numerous epidemiological studies have demonstrated that the consumption of brassica vegetables such as broccoli, red cabbage, and radish have health-promoting effects in consumers [6,7,8]. Growing evidence reveals that consuming young seedlings from these vegetables offers potential therapeutic benefits in diabetic management by eliminating predisposing factors [8,9]. For example, broccoli sprouts are known to rectify insulin resistance in type 2 diabetes, in addition to reducing side effects associated with the chronic condition [8]. Broccoli sprouts are rich in flavonoids and other polyphenolic compounds that can exert both anti-inflammatory and antioxidant activities, thereby protecting against diabetes [9]. Similarly, red cabbage offers therapeutic potential due to its high content of bioactive metabolites, including anthocyanins, flavonols, and glucosinolate [7]. Thus, among the target food products, broccoli and red cabbage sprouts are becoming major candidates in the search for diabetes remedies. Germination is a simple but effective technique to produce edible seeds with improved biological functions. Gan et al. asserted that germination can lead to accumulation of various bioactive components of edible seeds including vitamins, polyphenolic, and non-polyphenolic compounds through de novo synthesis of secondary metabolites [10]. It has been widely recognized that germinated seeds comprise a wide range of bioactive components that require an ongoing assessment to fully reveal their potential role in attenuating chronic illnesses.

Metabolomics is a new discipline in the area of “omics”. The technique allows simultaneous detection and characterization of metabolites synthesized in different metabolic processes. Untargeted metabolomics approaches screen a whole spectrum of metabolites in sample extracts and can be used to compare metabolite profiles of different samples. Owing to the diversity of plant metabolites and the underlying metabolic pathways involved in their synthesis, multiple metabolites can be produced during the germination process of edible seeds. Therefore, the first aim of this study was to investigate the alterations caused by germination on metabolite profiles of broccoli and red cabbage seeds using a UHPLC-ESI-QTOF-MS/MS-based untargeted metabolite profiling approach. The second objective was to assess the impact of germination on the biological activities of these seeds related to their antioxidant and antidiabetic potentials. To the best of our knowledge, this is the first time the untargeted metabolomics method has been used to compare the entire metabolite profile of broccoli and red cabbage seeds and sprouts. We also report for the first time the presence of diverse amino acids in broccoli and red cabbage seeds after germination. The study will contribute to the ongoing assessment of the potential therapeutic effects of germinated seeds in managing chronic conditions.

## 2. Materials and Methods

### 2.1. Plant Material Collection

**Figure 1:**
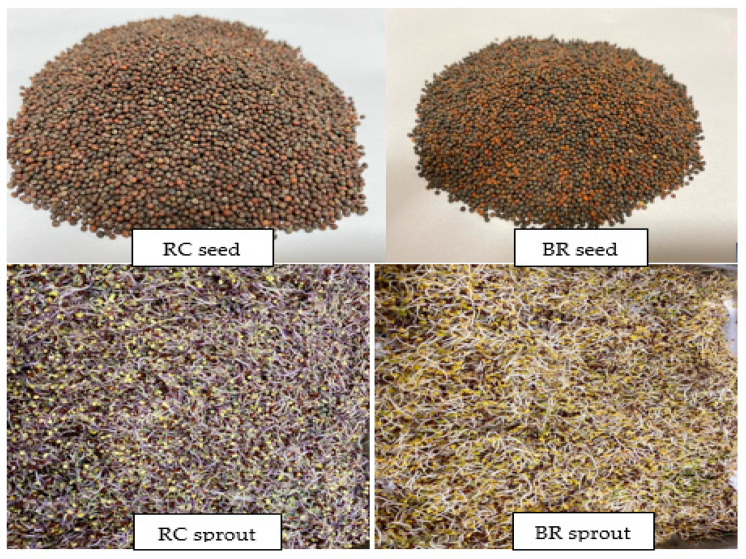
Pictures of raw seeds and sprouts provided by Charm-Chae-One, Ltd. (Jincheon, Chungbuk, Korea). RC, red cabbage; BR, broccoli.

Samples used in this experiment were obtained from Charm-Chae-One, Ltd. (Jincheon, Chungbuk, Korea), as shown in Figure 1. The sprouts were dried in an oven and the seeds and sprouted samples were pulverized using an electric mill to obtain a fine powder. Before extraction, the samples were kept at −20 °C.

### 2.2. Chemicals and Reagents

The liquid chromatography standards: ferulic acid and catechin were purchased from Sigma-Aldrich (Seoul, Korea). The α-glucosidase enzymes derived from Saccharomyces cerevisiae, pancreatic lipase, 4-Methylumbelliferone (4-MU), aminoguanidine (AG), Sulphuric acid, vanillin, soyasaponin B, orlistat, and acarbose were purchased from Sigma-Aldrich (Seoul, Korea). Sodium carbonate, methyl cellosolve, potassium phosphate dibasic, potassium phosphate monobasic, sodium dihydrogen phosphate, sodium phosphate dibasic sodium chloride, sodium citrate, 4-nitrophenyl α-d-glucopyranoside, Folin–Ciocalteu reagent, 2,2-diphenyl-1-picrylhydrazyl (DPPH), 2,2-azino-bis (3-ethylbenzothiazoline-6-sulfonic acid) diammonium salt (ABTS), 6-hydroxy2,5,7,8-tetramethylchromane-2-carboxylic acid (Trolox), ethanol, sodium hydroxide, and hydrochloric acid, sodium carbonate, and potassium per-sulfate were purchased from Sigma-Aldrich (Seoul, Korea). All the chemical reagents used were of analytical grade.

### 2.3. Preparation of Ethanol Extracts

The ethanol extracts were prepared using the method adopted by Pradeep and Sreerama [11] with some modifications. Briefly, 5 g of each sample was weighed and extracted with 70% ethanol (1:20 *w*/*v*) using an orbital shaker for 1 h at 50 °C. Following the centrifugation at 4000× *g* for 10 min, the supernatant was collected in dark bottles and the residue was re-extracted twice under the same set conditions. The final supernatant from each sample was combined and concentrated under vacuum at 40 °C followed by freeze-drying. The freeze-dried containing lyophilized solids obtained were kept at −20 °C and reconstituted in ethanol for further analysis.

### 2.4. Total Phenolic Compounds

Total phenolic content (TPC) was estimated using a 24-well microplate and ferulic acid as the standard according to the method described by Ainsworth and Gillespie, with some modifications [12]. In brief, 200 µL Folin–Ciocalteu reagent was added to 100 µL sample extracts or the standard or 95% (*v*/*v*) methanol as blank. The mixture was then vortexed followed by incubation at room temperature for 2 h. Then, 800 µL of 700 mM sodium carbonate was added to each of the mixtures and the absorbance was read using a SpectraMax i3 plate reader (Molecular Devices Korea, LLC, Seoul, Korea) at 765 nm. The TPC was calculated from the standard ferulic acid curve and expressed as a milligram of ferulic acid equivalents per 100 g of sample.

### 2.5. Total Flavonoid Content (TFC)

The total flavonoid content (TFC) of ethanol extracts was measured using a 24-well microplate according to the method described by Ofosu et al., with slight modifications [4]. In summary, using 250 µL sample extracts, the standard was pipetted into the wells of microplates followed by the addition of 75 µL NaNO_2_ (50 g L^−1^) and 1 mL distilled water. The mixture was allowed to settle for 5 min, then 75 µL AlCl_3_ (100 g L^−1^) was added. The reaction mixture was again allowed to settle for a while, then 500 µL of 1 M NaOH and 600 µL distilled water were added 6 min later. The absorbance was read at 510 nm using a Spectra-Max i3 plate reader (Molecular Devices Korea, LLC, Seoul, Korea) following 30 s of shaking. The TFC was calculated from catechin standard and expressed as milligram catechin equivalents per 100 g of sample (mg CE/100 g).

### 2.6. Total Saponin Content

The total saponin content (TSC) was analyzed according to the procedure described by Mendoza-Sánchez et al. [13]. Briefly, 100 µL of the sample extract was mixed with 1 mL of 72% sulfuric acid followed by the addition of 100 µL of 8% vanillin in ethanol. The mixture was incubated at a temperature of 60 °C for 20 min, cooled over cold water, then the absorbance was measured at 544 nm. The saponin content was obtained from the standard curve of soysaponin B and expressed as milligram soysaponin B equivalents per 100 g of sample.

### 2.7. Antioxidant Activities Assays

#### 2.7.1. DPPH Radical Scavenging Activity

The DPPH test was carried out according to the methods discussed by Li et al. [14], with few modifications. Briefly, 100 µM of freshly prepared DPPH radical solution (4 mg DPPH in 100 mL 95% *v*/*v* methanol) was mixed with 200 µL of sample extract or Trolox (concentration 1 mg/mL) in a 24-well microplate and incubated for 30 min in the dark at room temperature. The blank was performed following the procedure without extracts. The absorbance was read at 517 nm and the radical scavenging activities of the samples were expressed as percentage inhibition of DPPH by ethanol extracts (1 mg/mL) according to the following formula:

$$\mathrm{Inhibition}\;\left(\%\right)=\left(\frac{Ab-Aa}{Ab}\right)\times 100$$

where *Aa* is the absorbance value of the extracts or the standard, while *Ab* is the absorbance value of the blank sample. 

#### 2.7.2. ABTS Radical Scavenging Activity

ABTS assay was done according to the procedure described by Xiang et al. [15]. ABTS plus a stock solution was prepared by mixing an equivalent amount of 7 mM ABTS solution with 2.45 mM potassium persulfate solution and kept in the dark for 16 h at room temperature. The ABTS+ stock solution was constantly diluted until the absorbance of 0.70 at 734 nm was reached. Subsequently, 80 µL sample extract or Trolox (1 mg/mL) as added to 1 mL of the freshly prepared ABTS+ solution and absorbance was read at 734 nm. The results were expressed as percentage inhibition of ABTS using a similar formula to that described for DPPH assay above. From the Trolox standard curve, the antioxidant activity was also expressed as µmol Trolox equivalent/g DW.

### 2.8. Digestive Enzyme and Glycation Inhibition Assays

#### 2.8.1. α-Glucosidase Inhibitory Assay

The α-glucosidase inhibitory activity of the extracts and standard was done following procedures described by Sekhon-Loodu and Rupasinghe [16]. Briefly, 1 mg/mL of the extract was prepared in 10 mM potassium phosphate buffer (pH 6.8). Then, 100 µL extract (1 mg/mL) was pipetted into the microtiter plate. Next, 100 µL of freshly prepared a-glucosidase (0.5 U/mL) was then added followed by the addition of 300 µL of 10 mM potassium phosphate buffer (pH 6.8). The reaction mixture was incubated at 37 °C for 15 min before proceeding to the next stage. After the 15 min pre-incubation, 100 µL of 5 mM p-nitrophenol-α-d-glucopyranoside substrate was added and the final reaction mixture was incubated at 37 °C for a further 15 min. Finally, 400 µL of the stop solution (200 mM sodium carbonate) was added and the absorbance reading was taken at 405 nm using a SpectraMax i3 plate reader (Molecular Devices Korea, LLC, Seoul, Korea). A known antidiabetic drug (acarbose) mixed with enzyme, substrate, and buffer instead of inhibitors was used as a positive control. The sample blanks containing test sample, substrate, and buffer without α-glucosidase were also assayed. The percentage inhibition (%) of α-glucosidase by 1 mg/mL of the test sample was calculated according to the following formula:

$$\mathrm{Inhibition}\;\left(\%\right)=\left(\frac{AC-AS}{AC}\right)\times 100$$

where *AC* and *AS* are the absorbance recorded for the control and test samples respectively.

#### 2.8.2. Pancreatic Lipase Inhibition Assay

The assay used was described by Ofosu et al., whereby 4-MU oleate substrate was dissolved in methyl cellosolve and used for the assay [17]. Next, 50 µL (50 U/mL) of lipase dissolved in methyl cellosolve was pipetted into 50 µL of 1 mg/mL ethanolic extracts and standard (Orlistat) in a 24-well microtiter plate. The mixture was allowed to settle for 10 min at room temperature for the reaction to proceed. After 10 min, 100 µL of 1 mM 4-MU solution was added and incubated at room temperature (25 °C) for 30 min followed by the addition of 100 µL sodium citrate solution (0.1 M, pH 4.2) to stop the reaction. The amount of 4-methylumbelliferone released by hydrolysis of pancreatic lipase was quantified using a fluorescence reader at wavelengths of 355 nm and 460 nm. Experiments were carried out in triplicate and the percentage lipase inhibitory activity was calculated as shown below:

$$\mathrm{Lipase}\;\mathrm{Inhibition}\;\left(\%\right)=\left[1-\left(\frac{Ftest-Ftest\;blank}{Fcontrol-Fcontrol\;Blank}\right)\right]\times 100$$

where *Ftest* and *Ftest blank* represent the fluorescent readings for the test samples with and without the substrate 4-MU oleate respectively while *Fcontrol* and *Fcontrol blank* were the fluorescent readings of control with and without the substrate 4-MU oleate, respectively. The results were calculated as percentage lipase inhibition of the 1 mg/mL ethanolic extracts.

#### 2.8.3. Inhibition of AGE Formation

The AGE inhibition assay was done according to procedure discussed by Sekhon-Loodu and Rupa-Singhe, with few modifications [16]. Equal volumes (333 µL) of bovine serum albumin (BSA, 5.0 mg/mL), d-glucose (36 mg/mL), and negative control (without inhibitors); or the test samples; or aminoguanidine with established AGE formation inhibitor used as a positive control (concentrations 1.0 mg/Ml) were mixed in Eppendorf tubes. All the solutions were dissolved in a mixture of 0.2 M phosphate buffer saline (pH 7.4) and sodium azide (0.02% *w*/*v*). The mixtures were incubated at 37 °C for a week. Fluorescent AGEs in the mixtures were monitored on a microplate reader using 340 and 420 nm as the excitation and emission wavelengths, respectively. Experiments were carried out in duplicate and the percentage of the AGE inhibition was obtained as follows:

$$\mathrm{Inhibition}\;\left(\%\right)=\left[1-\left(\frac{Fluorescent\;of\;the\;test}{Fluorescent\;of\;control}\right)\right]\times 100$$

The results were calculated and expressed as percentage (%) inhibition of AGE formation by the ethanol extracts (1 mg/mL).

### 2.9. Ultra-High Performance Liquid Chromatography Quadrupole Time-of-Flight Mass Spectrometry (UHPLC-Q-TOF-MS/MS) of Bioactive Compounds

A UHPLC instrument (SCIEX Exion LC AD system, Framingham, MA, USA) was used to analyze the bioactive components of the samples according to the procedure described in the previous study carried out in our laboratory [4]. Briefly, the UHPLC SCIEX Exion LC AD system was fitted with various components, such as a controller, AD auto sampler, and photodiode array (PDA) detector. The analytical column was composed of a 100 mm × 3 mm Accucore C18 column. Then, 10 µL of the sample was injected using an autosampler and the sample was eluted into the column with a binary mobile phase consisting of various components, denoted as A (water containing 0.1% formic acid) and B (methanol). A flow rate of 0.4 mL/min was employed in this analysis. A 25 min linear gradient programmed according to the previous values reported by Ofosu et al. [4] was used. The compounds in the seeds and sprouts were identified by relating retention time (RT) and UV spectra information and confirmed by UHPLC-Q-TOF-MS2 (SCIEX, Framingham, MA, USA).

### 2.10. Statistical Analysis

The data analysis was done using Graphpad Prism 8.0 (GraphPad Software, San Diego, USA). The differences in mean values among different extracts of red cabbage and broccoli fractions were determined using one-way analysis of variance (ANOVA) followed by Tukey’s test at the significance level of *p* < 0.05. The results of the analysis are presented as means ± standard deviation (SD).

## 3. Results

### 3.1. TPC, TFC, and TSC of Ethanol Extracts

The TPC, TFC, and TSC values of ethanol extracts of raw seed and sprout extracts of broccoli and red cabbage were compared. The results are shown in Table 1. The TPC values of the red cabbage seeds and sprouts were 425.3 ± 25.52 and 393.7 ± 0.06 GAE mg/100 g DW, respectively; while the values for broccoli seeds and sprout extracts were 480.4 ± 19.13 and 385.4 ± 13.66 GAE mg/100 g DW, respectively. On the other hand, the levels of TFC in red cabbage seeds and sprouts were 215.6 ± 0.16 and 209.9 ± 0.03 CE/100 g, respectively. The TFC of broccoli sprout was 206.9 ± 0.02, while that of the raw broccoli seed was 216.9 ± 0.38 mg CE/100 g. The total saponin content (TSC) was calculated as soysaponin B equivalent values. TSC levels in the extracts ranged from 15.8 to 27.0 mg soysaponin B equivalent/100 g DW). BR sprout had the highest TSC at 27.0 ± 2.1 mg/100 g, which was significantly different from broccoli seeds, red cabbage seeds, and red cabbage sprouts, with values of 18.6 ± 3.90, 17.8 ± 3.90, and 15.8 ± 2.75 mg/100 g, respectively. Thus, it was concluded that the germination effects on the TFC, TPC, or TSC values of seeds depends on the type of seed species used. 

### 3.2. Antioxidant Capacity of Ethanol Extracts

**Figure 2:**
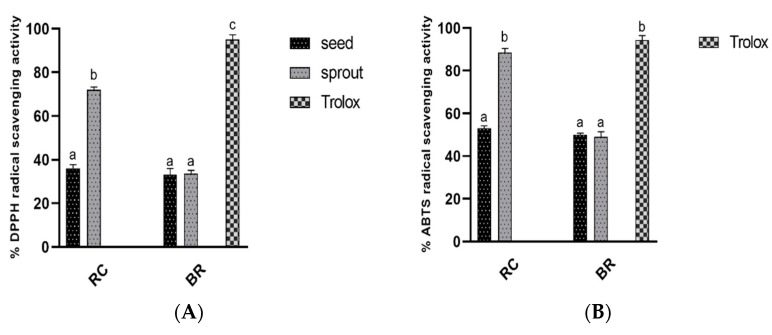
Antioxidant capacity levels of 1 mg/mL ethanol extracts of red cabbage and broccoli (seeds and sprouts): (**A**) percentage 2,2′-diphenyl-1-picrylhydrazyl (DPPH) radical scavenging activity values; (**B**) 2,2′-Azino-bis (3-ethylbenzothiazoline-6-sulfonate. BR, broccoli; RC, red cabbage.

The antioxidant capacity values of the extracts as measured by DPPH and ABTS are presented in Figure 2A,B as percentages and In Table 2 as micromole Trolox equivalent values. Broccoli seed extract had the lowest DPPH radical scavenging activity, with an inhibitory value of 31.34 ± 2.43%, which was not significantly different from the values for broccoli sprout (33.32 ± 2.41%) and red cabbage seed (34.99 ± 2.31%) (Figure 2A). Trolox (1 mg/mL), a standard antioxidant compound, showed the highest DPPH scavenging activity of 94.48 ± 2.43%, which was significantly higher than the value for red cabbage sprout extract (71.5 ± 2.51%). Consistent with DPPH radical scavenging activity, broccoli sprout, broccoli seed, and red cabbage seed had significantly lower ABTS radical scavenging activity levels, with percentage values of 50.04 ± 1.30%, 51.04 ± 1.21%, and 53.6 ± 2.43%, respectively, in comparison to red cabbage sprout (88.53 ± 2.10%) and Trolox (94.4 ±1.3%). However, there was no significant difference in the ABTS radical scavenging activity of red cabbage sprout extracts and Trolox (*p* < 0.05). Notably, ABTS values were relatively higher than DPPH values in all of the tested samples. The antioxidant activities were also expressed as micromole Trolox equivalent per gram of dry weight (µmol TAE, DW), as shown in Table 2. The findings reveal that germination may lead to a sharp increase in the antioxidant capacity of red cabbage making sprouted seeds, which are more appropriate as antioxidant ingredients compared to the raw seeds.

### 3.3. Antidiabetic Activity In Vitro

#### Alpha-Glucosidase, Pancreatic Lipase, and AGE Inhibitory Formation Activities

**Figure 3:**
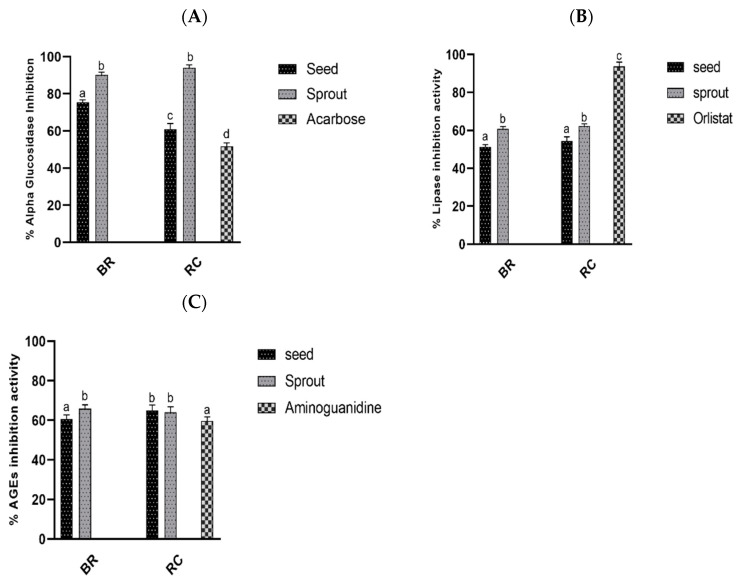
Antidiabetic activity in vitro of 1 mg/mL ethanol extracts: (**A**) percentage α-glucosidase inhibitory activity; (**B**) percentage pancreatic lipase inhibitory activity; (**C**) percentage AGE formation inhibitory activity. BR, broccoli; RC, red cabbage.

To assess the antidiabetic potential of the sprout and seed extracts, we analyzed the ability to inhibit digestive enzymes and AGE formation by 1 mg/mL of the extracts. The concentration used was 1 mg/mL for all samples and standards. The results are expressed as percentage inhibition of the ethanol extracts. (Figure 3A–C); Figure 3A, percentage α-glucosidase inhibitory activity; Figure 3B, percentage pancreatic lipase inhibitory activity; Figure 3C, percentage AGE formation inhibitory activity of the extracts. Red cabbage sprout exhibited the highest inhibition of α-glucosidase, with a percentage value of 93.11 ± 2.30%. However, this was not significantly different from broccoli sprouts (91.32 ± 2.43%) (*p* < 0.05). Nonetheless, the acarbose standard compound (1 mg/mL) showed the lowest inhibition of α-glucosidase, with a percentage value of 51.32 ± 2.38%, followed by red cabbage seed (60.12 ± 1.40%) and broccoli seed extracts (75.33 ± 3.11%) (Figure 3A). On the other hand, the antipancreatic lipase assay revealed that broccoli sprout and red cabbage ethanol extracts exhibited the highest inhibitory activity levels against the lipase enzyme (61.66 ± 2.79% and 60.19 ± 2.73%, respectively), which were significantly less effective than prescribed standard orlistat (91.21 ± 2.39%) but higher compared to red cabbage and broccoli seed extracts at 52.74 ± 2% and 50.99 ± 2.1%, respectively. Moreover, the AGE formation inhibitory activity levels of the extracts was recorded according to Figure 3C. Broccoli sprout ethanol extract exerted the highest inhibitory activity against AGE formation, with a percentage value of 65.4 ± 2.76%, which did not differ significantly from the values of red cabbage seeds (64.13 ± 2.40%) or red cabbage sprouts (63.15 ± 2.3%) (*p* < 0.05). The standard aminoguanidine exerted the lowest activity against AGE formation (59.19 ± 2.90%), followed by broccoli seed (60.37 ± 2.61%). In general, germination significantly enhanced the AGE inhibitory activity of broccoli but did not result in significant changes in the AGE inhibitory activity of red cabbage.

### 3.4. Analyses of Metabolites Using UHPLC-Q-TOF-MS2

**Figure 4:**
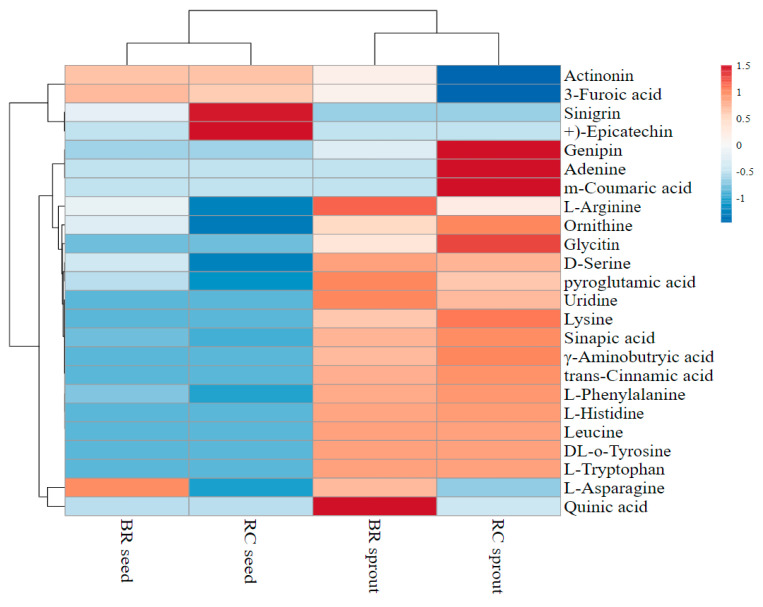
Heatmap plot showing the profiles and levels of metabolites identified in broccoli and red cabbage (seed and sprout) extracts.

The untargeted assessment of the chemical compositions of red cabbage and broccoli seeds and sprout extracts was performed using UHPLC-Q-TOF-MS2. The compounds were identified based on their health-promoting effects, particularly their antidiabetic and antioxidant activities. The characterization of the metabolites was accomplished by comparing retention time (RT) values with available authentic database standards, as confirmed by UHPLC-Q-TOF-MS2. The metabolites were tentatively identified by comparing spectral data with spectral evidence from the literature and were crosschecked with other data available in in Metlin database (https://isometlin.scripps.edu) and Metabolomics Workbench database (https//www.metabolomicsworkbench.org). As shown in Table 3, Table 4, Table 5 and Table 6, the identified metabolites are listed along with their peak numbers, retention times, molecular weights of [M−H]^-^(*m*/*z*), molecular formulas, and MS/MS product ions (% abundance of ion fragments). The UHPLC-Q-TOF-MS2 tentatively characterized a total of twenty-four (24) compounds in the four samples. The heatmap was developed using the peak areas of the metabolites obtained from the UHPLC-Q-TOF-MS2 analysis (Figure 4). The peak areas of metabolites representing their concentration or relative levels in extracts are shown in Supplementary Table S1. Accordingly, twenty (20), ten (10), twenty-one (21), and eight (8) metabolites were tentatively identified in the ethanol extracts of broccoli sprout, broccoli seed, red cabbage sprout, and red cabbage seed, respectively (Table 3, Table 4, Table 5, and Table 6 respectively). Red cabbage sprout extract had the highest concentration of metabolites (Table 5). Amino acids constituted the majority of the compounds identified in the extracts. Most of the identified compounds were not present in raw seed extracts but were found in sprout extracts. However, some of the metabolites were also lost during germination process. Therefore, germination either enhanced the content of metabolites, synthesized new compounds, or led to loss of some metabolites in the seeds.

## 4. Discussion

Phenolic compounds are important secondary metabolites that demonstrate potential health benefits when consumed. These compounds may act as natural antioxidants attenuating the side effects of free radicles in chronic conditions in humans [18]. It was, therefore, essential to determine their levels in this research. Past studies have reported on changes of the TPC content when seeds were germinated [19,20,21]. Previous studies have indicated that germination can gradually lead to TPC accumulation in germinated edible seeds by stimulating enzymes such as phenylalanine ammonia lyase (PAL), which plays key roles in the de novo pathways [10,19,22]. However, the current study showed contrasting results, revealing that germination either had no effect or significantly decreased TPC levels in sprouts. For instance, germination did not significantly affect TPC levels in red cabbage seeds but significantly reduced their levels in broccoli seeds from 480.4 ± 19.13 to 385.4 ± 13.66 mg FAE/100 g DW. The variation between these findings and those reported in previous studies may be due to factors such as the type of seed variety and germination conditions used [23]. Nevertheless, our results were consistent with those reported for red cabbage [23] and mung bean [24,25], indicating that germination can actually lead to either increased or decreased levels or may not significantly affect TPC levels in the germinated seeds. 

Flavonoids are the major types of phenolic compounds, which are useful in protecting against diabetes in humans, in addition to playing various key roles, such as protecting the plant against environmental stress [22]. Earlier, it was reported that germination improved TFC levels in broccoli [20] and red cabbage [21]. In contrast to these reports, our study revealed that germination significantly decreased the levels of TFC in red cabbage from 215.7 ± 0.16 to 209.9 ± 0.03 mg CE/100 g. Similarly, TFC levels in broccoli seeds significantly decreased upon germination from 216.9 ± 0.38 mg CE/100 g in raw seeds to 206.9 ± 0.02 mg CE/100 g in sprouts. There were no significant differences in the levels of TFC in broccoli seed (216.9 ± 0.38 mg CE/100 g) and red cabbage seed (215.6 ± 10.2 mg CE/100 g) extracts. Likewise, no significant difference was observed in the TFC levels of broccoli sprout extract (206.9 ± 10.2 mg CE/100 g) and red cabbage sprout extract (209.9 ± 10.2 mg CE/100 g). Factors that may lead to decreased TPC and TFC levels in seeds upon germination may include leaching of soluble phenolic compounds into the soaking water and the breakdown of bound phenolic compounds to form other compounds during germination.

Saponins are naturally occurring surface-active glycosides in plants. Saponin have been shown to exert potent glycemic control and prevent disorders associated with hyperglycemia. According to Elekofehinti, the antidiabetic activity of saponin is mainly due to its ability to induce insulin production from the pancreas [26]. Saponins could also be involved in various therapeutic pharmacological activities in diabetic conditions, including inhibition of AGE formation and oxidative stress, as well as transformation of growth factor β1 (TGFβ1), thereby preventing the development of diabetic nephropathy [26]. Our study revealed the presence of saponin in both broccoli and red cabbage. However, while germination did not significantly impact their levels in red cabbage (*p* < 0.05), the process sharply enhanced the TSC levels in broccoli sprouts, as described in Table 1. The literature also suggests that there is a notable impact on saponin content upon germination of seeds [27,28]. The decreases in the saponin levels observed for red cabbage sprouts in this work is in agreement with reports that germination process reduces these compounds in seeds [28]. Additionally, the observation for broccoli sprouts that germination actually improved saponin levels in seeds were in agreement with the results reported by Ayet, who noted lower levels of saponin and phytic acid during germination of seeds [27]. It is known that germination reduces antinutritive factors in plants, however this has little effect on the compounds involved in the plants’ defense mechanisms [27]. Therefore, an increase or decrease in saponin content in seeds during germination could be explained by the implication of these compounds in the plant defense system.

Over decades, studies have supported the role of oxygen free radicals as mediators in the development of diabetic complications [29]. Developing a natural antioxidant that can inhibit the effects of the free radicle is considered a milestone in diabetic management [29]. The antioxidant activity levels of raw seeds and sprouts were analyzed in this study. Even though germination did not significantly affect the antioxidant ability of broccoli seeds, it led to a sharp increase in the antioxidant capacity of red cabbage (shown in Figure 2A,B and Table 2). The current findings were consistent with those reported by Vale et al., who described increased antioxidant activities for red cabbage samples germinated under light [30]. Therefore, germination can act as a simple and effective method to improve the antioxidant activities of edible seeds.

The hydrolyzing enzymes such as α-glucosidase and pancreatic lipase are important in diabetes control. While α-glucosidase catalyzes the cleavage of the absorbable sugars from disaccharides and oligosaccharides, increasing the blood glucose levels, pancreatic lipase hydrolyzes lipids, leading to fat accumulation in the pancreas [31,32,33]. Hence, the primary target to control postprandial blood glucose levels and lipid accumulation is to develop compounds that can competitively inhibit digestive enzymes, thereby reducing the hydrolysis of carbohydrates and lipids in the body. In the past, commercially prepared α-glucosidase and lipase inhibitors such as acarbose and orlistat have been used to control the activity of these enzymes in the body. Nevertheless, these compounds are associated with gastrointestinal side effects, limiting their use [34]. The production of natural inhibitors of α-glucosidase and pancreatic lipase from plant-based sources may provide an alternative means to treat diabetes or manage it without involving synthetic inhibitors. Within the current collection of foods, germinated seeds have demonstrated an effective source for an attractive strategy to control postprandial hyperglycemia and excessive accumulation of lipids in the body [31]. The inhibition of α-glucosidase, pancreatic lipase activities, and AGE formation by ethanol extracts of broccoli and red cabbage were investigated in this study (Figure 3A–C). Germinating red cabbage and broccoli seeds led to increased α-glucosidase inhibition by 33% and 16%, respectively. Similar observations were made for pancreatic lipase inhibition activities, for which increases of about 7% and 10% were observed for red cabbage and broccoli, respectively, upon germination. 

Simple sugars such as glucose can non-enzymatically undergo a reactive process with the amino groups of proteins, nucleic acids, and fats, leading to the formation of glycated senescent macromolecules known as AGEs. The formation of AGEs progresses faster under inflammatory and hyperglycemic conditions, leading to loss of the integrity of the macromolecules [5]. Earlier reports indicated that plant extracts were able to block the expression of RAGE pathways, thereby reducing the effects of AGEs in vivo [35]. Germination significantly improved the AGE activities of broccoli sprouts but had little impact on the activities of red cabbage (Figure 3C). Maeda et al. also reported similar results for the broccoli sprouts with higher sulforaphane contents [36]. The phytochemicals, including alkaloids, flavonoids, saponins, and steroids, are responsible for the antidiabetic activities in brassica vegetables [37].

The rich bio-composition of key secondary metabolites within the Brassicaceae vegetables covers many compounds that can be extracted in alcohol solutions. Most of these metabolites are recognized as phytochemicals, which are known for their roles in the prevention of chronic diseases, as well as other health-promoting benefits. The current study used UHPLC-Q-TOF-MS2 to analyze the metabolites in the broccoli and red cabbage extracts. In the sprout–raw seed comparison, 16 metabolites were upregulated and 4 were downregulated in broccoli seeds after germination (Figure 4). Similarly, the contents of 17 metabolites were enhanced while the levels of 4 metabolites were reduced by the germination process in red cabbage seeds. Correspondingly, the UHPLC-Q-TOF-MS2 analysis showed that the change trends for metabolites in seed triggered by the germination process varied. For example, germination caused 0.75-, 0.64-, 1.52-, and 5.53-fold increases in the levels of ornithine, l-arginine, d-serine, and l-phenylalanine, respectively, in broccoli sprouts; while l-asparagine was decreased by 5.53-fold. Similarly, l-arginine and l-asparagine were increased by 1.50- and 0.24-fold in red cabbage sprouts, respectively. Moreover, some of the metabolites that were previously not present in seeds were synthesized in the germination process. 

Amino acids, other protein-derived compounds, and phenolic compounds were the prominent metabolites observed in this study. Nonetheless, amino acids were undisputedly the most detected and identified compounds in the extracts. By comparing the spectral data with those available in standard databases and other literature reports, the metabolites were tentatively identified (Table 3, Table 4, Table 5 and Table 6). For instance, amino acids with deprotonated [M−H]^-^ molecules at *m*/*z* values of 145.098, 154.062, 104.035, 131.046, 180.067, 130.087, 164.072, 203.083, and 445.115 were tentatively identified as lysine, l-histidine, d-serine, l-asparagine, dl-o-tyrosine, leucine, l-phenylalanine, l-tryptophan, and glycitin, respectively, by comparing their masses (*m*/*z*) with those in the mass spectral libraries XCMS Online (Metlin) and Metabolomics Workbench and further crosschecked with the *m*/*z* data reported by Hanhineva et al. [38]. Fourteen (14) and fifteen (15) amino acids were tentatively identified in broccoli and red cabbage sprout extracts, respectively, ( Table 3; Table 5). l-Arginine, pyroglutamic acid, and l-asparagine were the only amino acids present in the red cabbage seed extract (Table 6), while ornithine, l-asparagine, pyroglutamic acid, lysine, d-serine, and l-phenylalanine were the prominent amino acids in broccoli seed extract (Table 4). Amino acids are important in plants as well as in humans. Some of these amino acids have already been shown to have positive physiological importance in the body, such as participating in the synthesis of macromolecules. However, plasma-free amino acid profiles have been associated with increased risks of diabetes type 2 [39]. Therefore, amino acids have the potential to be used as biomarkers for assessing diabetes risk, as well as in monitoring strategies designed to reduce such risks [39]. In addition to enhancing their levels, germination processes also resulted in the synthesis of new amino acids, including l-histidine, l-tryptophan, leucine, and lysine, which were previously not present in raw seed samples. Consequently, germination was an essential process needed to improve the concentrations of various amino acids in edible seeds. Furthermore, γ-aminobutryic acid, the major non-protein amino acid identified in the germinated broccoli and red cabbage sprout extracts deprotonated at the [M−H]^-^*m*/*z* value of 102.056 was confirmed by the product ions of *m/z* 102 (100%), 101 (5%), using the Metlin database (https://isometlin.scripps.edu). GABA is a known neurotransmitter in the body, as well as an antioxidant, antidiabetic, and antiobesity compound, among other biological functions [40]. A similar identification method was applied to tentatively identify other protein-derived compounds that were deprotonated at *m*/*z* 243.062 and 134.047, namely uridine and adenine, respectively. The uridine and adenine are nucleic acid components that were only found in the sprouted seed extracts.

Previous studies have shown that glucosinolates are prominent groups of compounds exerting beneficial health effects in brassica vegetables [41]. Glucosinolates are key metabolites involved in controlling obesity, diabetes, and many other cardiovascular diseases [38]. In the present study, sinigrin was identified as the only glucosinolate at *m*/*z* 358.027 present in broccoli and red cabbage seed extracts after comparison with MS spectra data from the literature [42]. In terms of phenolic profile subclasses, flavanol and phenolic acids were found in the extracts. Compound C_15_H_14_O_6_ exhibited a deprotonated [M−H]^-^ molecule at *m*/*z* 289.0721, with fragmented ions at *m*/*z* values of 245 and 151, with the most intense value of 289 being detected in raw broccoli seeds. The compound C_15_H_14_O_6_ was tentatively identified as (+)-epicatechin via comparison with MS spectral data in the literature [43]. (+)-Epicatechin and other catechin derivatives have been reported to exert powerful antioxidant and anti-inflammatory activities in diabetic conditions [44].

Phenolic acids and other organic acids were widely present in broccoli and red cabbage extracts. Compounds with deprotonated [M−H]^-^ molecules at *m*/*z* 191.056, 223.061, 163.040, and 111.009 were identified as quinic acid, sinapic acid, m-coumaric acid, and 3-furoic acid, respectively, using the method described above and via crosschecking with figures reported in the literature by Hong et al. [45]. Phenolic acids play key roles in various biological processes in humans, in addition to their roles in the synthesis of other phenolic compounds in plants. For instance, quinic acid derivatives are polyphenol-rich compounds consisting of a large group of esters synthesized from quinic acid and one or more phenylpropanoic acid, such as sinapic acid, coumaric acid, and cinnamic acid [46]. Quinic acid and its derivatives have been reported to exert antidiabetic properties by alleviating structural degeneration in the pancreas, liver, and kidneys [46]. Moreover, sinapic acid was also reported as a strong antioxidant compound in vitro [47]. Interestingly, sinapic acid and quinic acid were found at lower levels in seed extracts compared to sprout extracts. The absence of certain antioxidant and antidiabetic phytochemicals or their lower concentrations in raw seed explains why germinated samples exhibited higher activities compared to raw samples. All other remaining metabolites were tentatively identified using the methods described above.

We reported for the first time the synthesis of diverse amino acids, including l-arginine, d-serine, and l-phenylalanine, as well as lysin, in broccoli and red cabbage seeds upon germination. These findings are unique in revealing the role of germination in enhancing not only phenolic compounds but also amino acid profiles in edible seeds. The current findings confirm the results of previous studies showing that germination can significantly alter the metabolite compositions of edible seeds, in addition to enhancing their functional properties. 

## 5. Conclusions

The present study offers new insights into the potential impacts of germination on the antioxidant ability, antidiabetic ability, and metabolite composition of red cabbage and broccoli seeds. Germination did not affect or significantly reduce the TPC or TFC levels of the seeds. However, it improved the saponin levels in broccoli sprouts but decreased their levels in red cabbage sprouts. The in vitro assessment of the antidiabetic potential of the extracts revealed that sprout extracts demonstrated better potential as antioxidant, α-glucosidase, and pancreatic lipase inhibitors compared to raw seeds, indicating their potential to retard the development of diabetes by reducing the effects of free radicals and hydrolyzing enzymes in the body. Furthermore, unlike red cabbage sprouts, germination significantly enhanced the AGE formation inhibitory properties of broccoli extracts, thereby improving their ability to lessen the deleterious consequences of AGEs formed in diabetic conditions. Moreover, a total of 24 metabolites were characterized in the extracts using UHPLC-QTOF-MS/MS. Germination not only enhanced the levels of metabolites but also synthesized new compounds in edible seeds. Amino acids and phenolic compounds were the most improved metabolites in the germination process. The results from this study are useful for the development of functional foods from germinated seeds to help in the prevention and management of diabetes and other illnesses associated with oxidative stress. Further studies using animal models are needed to confirm the antidiabetic ability of broccoli and red cabbage seeds and sprouts in vivo.

## Figures and Tables

**Table 1:** Total phenolic content (TPC), total flavonoid content (TFC), and total saponin content (TSC) values of red cabbage and broccoli seeds and sprouts.

Extracts	TPC (mg Ferulic Acid Equivalent/100 g, DW)	TFC (mg Catechin Equivalent/100 g, DW)	TSC (mg Soysaponin B Equivalent/100 g, DW)
RC Seed	425.3 ±25.52 ^a^	215.6 ± 0.16 ^a^	17.8 ± 3.90 ^a^
RC sprout	393.7 ± 0.06 ^a^	209.9 ± 0.03 ^b^	15.8 ± 2.75 ^a^
BR seed	480.4 ± 19.13 ^b^	216.9 ± 0.38 ^a^	18.6 ± 3.90 ^a^
BR sprout	385.4 ± 13.66 ^a^	206.9 ± 0.02 ^b^	27.0 ± 2.1 ^b^

Results are expressed as means ± SD. Different superscripts within each column denote significant differences (*p* < 0.05). DW, dry weight sample.

**Table 2:** Antioxidant activity (µmol Trolox equivalent/g, DW).

Extracts	DPPH (µmol Trolox Equivalent/g, DW)	ABTS(µmol Trolox Equivalent/g, DW)
RC Seed	4.76 ± 0.43 ^a^	15.15 ± 0.43 ^a^
RC sprout	13.89 ± 0.51 ^b^	19.91± 0.10 ^b^
BR seed	3.86 ± 0.43 ^a^	15.06 ± 0.21 ^a^
BR sprout	4.34 ± 0.41 ^a^	14.89 ± 0.30 ^a^

Radical scavenging activity levels of 2,2′-diphenyl-1-picrylhydrazyl (DPPH) and 2,2′-azino-bis (3-ethylbenzothiazoline-6-sulfonate (ABTS) expressed as micromole Trolox equivalent per gram, DW.

**Table 3:** Metabolites identified in the ethanol extracts of broccoli sprouts using UHPLC-Q-TOF-MS2. RT, retention time.

Peak No.	RT Per Min	Molecular Weight	[M−H]^-^(*m*/*z*)	Molecular Formula	MS/MS (%) Abundance	Compound Identified
1	0.69	146.10579	145.098	C_6_H_14_N_2_O_2_	145 (100%)	Lysine
2	0.7	155.06990	154.0621	C_6_H_9_N_3_O_2_	93 (65%), 67 (20%), 137 (15%)	l-Histidine
3	0.7	174.112	173.104	C_6_H_14_N_4_O_2_	173 (100%)	l-Arginine
4	0.71	132.08994	131.0823	C_5_H_12_N_2_O_2_	131 (52%), 88 (100%)	Ornithine
5	0.78	105.04267	104.035	C_3_H_7_NO_3_	74 (98%)	d-Serine
6	0.78	132.05364	131.0461	C_4_H_8_N_2_O_3_	58 (45%), 113(47%)	l-Asparagine
7	0.81	103.06344	102.0557	C_4_H_9_NO_2_	102 (100%), 101 (5%)	γ-Aminobutryic acid
8	0.91	192.06378	191.056	C_7_H_12_O_6_	85 (100%) 191 (20%)	quinic acid
9	1.05	290.02528	289.0177	C_5_H_7_NO_3_	289 (100%), 72 (34%),	pyroglutamic acid
10	1.22	244.070	243.062	C_9_H_12_N_2_O_6_	110 (100%), 82(63%), 122(40%), 66(38%)	Uridine
11	1.23	181.07420	180.0665	C_9_H_11_NO_3_	93 (100%), 119 (100%), 163 (99%)	dl-o-Tyrosine
12	1.23	112.01616	111.0084	C_5_H_4_O_3_	65 (100%), 70 (23%)	3-Furoic acid
13	1.25	131.09475	130.0871	C_6_H_13_NO_2_	130 (100%)	Leucine
14	2.98	165.07919	164.0715	C_9_H_11_NO_2_	77 (100%) 103 (100%)	l-Phenylalanine
15	5.9	204.09023	203.0825	C_11_H_12_N_2_O_2_	116 (100%), 142 (28%)	l-Tryptophan
16	2.98	148.05232	147.0451	C_9_H_8_O_2_	62 (98%), 77 (20%)	Trans cinnamic acid
17	13.33	385.25852	384.2509	C_19_H_35_N_3_O_5_	111(37%), 180 (20%), 224 (39%)	Actinonin
18	15.91	226.08431	225.0769	C_11_H_14_O_5_	77 (59%), 121(40%)	Genipin
19	16.75	224.06879	223.061	C_11_H_12_O_5_	121 (78%), 149 (59%), 164 (47%), 223 (19%), 193 (11%), 208 (4%)	Sinapic acid
20	16.78	446.12227	445.1146	C_22_H_22_O_10_	189 (97%), 121 (65%), 188 (44%)	Glycitin

**Table 4:** Metabolites identified in the ethanol extracts of raw broccoli seeds using UHPLC-Q-TOF-MS2. RT, retention time.

Peak No.	RT Per Min	Molecular Weight	[M−H]^-^(*m*/*z*)	Molecular Formula	MS/MS (%) Abundance	Compound Identified
1	0.7	132.090	131.0823	C_5_H_12_N_2_O_2_	131 (52%), 88 (100%)	Ornithine
2	0.7	174.112	173.104	C_6_H_14_N_4_O_2_	173 (100%)	l-Arginine
3	0.77	132.054	131.0459	C_4_H_8_N_2_O_3_	53 (100%), 70 (99%)	l-Asparagine
4	0.77	105.043	104.035	C_3_H_7_NO_3_	70 (100%)	d-Serine
5	1.05	290.02528	289.018	C_5_H_7_NO_3_	289 (100%), 72 (34%),	pyroglutamic acid
6	1.12	359.035	358.027	C_10_H_17_NO_9_S_2_	74 (53%), 95 (42%), 96 (100%), 274 (3%)	Sinigrin
7	1.22	112.016	111.008	C_5_H_4_O_3_	65 (100%), 70 (23%)	3-Furoic acid
8	2.98	165.079	164.071	C_9_H_11_NO_2_	103 (100%)	l-Phenylalanine
9	13.33	385.258	384.251	C_19_H_35_N_3_O_5_	111(37%), 180 (20%), 224 (39%)	Actinonin
10	16.75	224.069	223.061	C_11_H_12_O_5_	164(62%), 149 (59%), 121 (30%),	Sinapic acid

**Table 5:** Metabolites identified in the ethanol extracts of red cabbage sprouts using UHPLC-Q-TOF-MS2. RT, retention time.

Peak No.	RT Per Min	Molecular Weight	[M−H]^-^(*m*/*z*)	Molecular Formula	MS/MS (%) Abundance	Compound Identified
1	0.69	146.1058	145.098	C_6_H_14_N_2_O_2_	145 (100%)	Lysine
2	0.7	155.070	154.062	C_6_H_9_N_3_O_2_	81 (100%), 93(80%), 67(62%)	l-Histidine
3	0.7	174.112	173.104	C_6_H_14_N_4_O_2_	173 (100%)	l-Arginine
4	0.77	105.043	104.035	C_3_H_7_NO_3_	70 (100%)	d-Serine
5	0.78	132.054	131.046	C_4_H_8_N_2_O_3_	58 (100%), 70 (100%)	l-Asparagine
6	0.8	132.090	131.082	C_5_H_12_N_2_O_2_	131 (52%), 88 (100%)	Ornithine
7	0.8	103.063	102.056	C_4_H_9_NO_2_	102 (100%), 101 (5%)	γ-Aminobutryic acid
8	0.93	135.055	134.047	C5H5N5	107 (66%), 92 (64%)	Adenine
9	0.91	192.064	191.056	C_7_H_12_O_6_	85 (100%) 191 (20%)	Quinic acid
10	1.05	290.02528	289.0177	C_5_H_7_NO_3_	289 (100%), 72 (34%),	pyroglutamic acid
11	1.22	181.074	180.067	C_9_H_11_NO_3_	119 (100%), 93 (33%), 163 (30%)	dl-o-Tyrosine
12	1.22	244.070	243.062	C_9_H_12_N_2_O_6_	110 (100%), 82(63%), 122(40%), 66(38%)	Uridine
13	1.25	131.09475	130.0871	C_6_H_13_NO_2_	130 (100%)	Leucine
14	2.97	165.079	164.072	C_9_H_11_NO_2_	103 (100%), 147 (100%), 72 (99%)	l-Phenylalanine
15	2.97	148.053	147.045	C_9_H_8_O_2_	62 (100%)	Trans cinnamic acid
16	5.9	204.090	203.082	C_11_H_12_N_2_O_2_	116 (100%), 142 (28%)	l-Tryptophan
17	13.32	385.259	384.251	C_19_H_35_N_3_O_5_	112 (59%), 93 (39%), 207 (16%)	Actinonin
18	15.82	164.0481	163.040	C_9_H_8_O_3_	117 (3%),65 (2%)	m-Coumaric acid
19	15.9	226.085	225.077	C_11_H_14_O_5_	123 (100%), 119 (15%)	Genipin
20	16.75	224.069	223.061	C_11_H_12_O_5_	149 (59%), 164 (58%), 121 (30%), 193 (20%)	Sinapic acid
21	16.78	446.122	445.115	C_22_H_22_O_10_	189 (97%), 121 (65%), 188 (44%)	Glycitin

**Table 6:** Metabolites identified in the extracts of raw cabbage seeds using UHPLC-Q-TOF-MS2. RT, retention time.

Peak No.	RT Per Min	Molecular Weight	[M−H]^-^(*m*/*z*)	Molecular Formula	MS/MS (%) Abundance	Compound Identified
1	0.7	174.112	173.104	C_6_H_14_N_4_O_2_	173 (100%)	l-Arginine
2	0.77	132.054	131.046	C_4_H_8_N_2_O_3_	58 (100%), 70 (100%)	l-Asparagine
3	1.05	290.02528	289.0177	C_5_H_7_NO_3_	289 (100%), 72 (34%),	pyroglutamic acid
4	1.12	359.035	358.028	C_10_H_17_NO_9_S_2_	358 (10), 116 (5%)	Sinigrin
5	1.22	112.016	111.009	C_5_H_4_O_3_	65 (100%), 70 (23%)	3-Furoic acid
6	13.35	385.2586	384.251	C_19_H_35_N_3_O_5_	112 (23%), 150 (17%), 224 (17%)	Actinonin
7	14.17	290.0796	289.072	C_15_H_14_O_6_	289 (10% /245 (8%) 151 (8) 97	+) -Epicatechin
8	16.75	224.069	223.061	C_11_H_12_O_5_	149 (59%), 164 (58%), 121 (30%), 193 (20%)	Sinapic acid

## Data Availability

Not applicable.

## Supplementary Materials

The following are available online at https://www.mdpi.com/article/10.3390/antiox10060852/s1, Table S1: Peak areas of metabolites representing their concentration or relative levels in extracts used to develop heatmap plot.

## Abbreviations

ABTS	2-azino-bis (3-ethylbenzothiazoline-6-sulfonic acid) diammonium salt
AGEs	advanced glycation end products
BR	broccoli
CE	catechin equivalent
DPPH	2, 2′-diphenyl-1-picrylhydrazyl
DW	dry weight
FAE	ferulic acid equivalent
RC	red cabbage
RAGE	receptor for advanced glycation end products
RT	retention time
TPC	total phenolic content
TSC	total saponin content
TFC	flavonoid content
