# Supplementary material for: UHPLC-ESI-QTOF-MS/MS Metabolite Profiling of the Antioxidant and Antidiabetic Activities of Red Cabbage and Broccoli Seeds and Sprouts
Source: Antioxidants (Basel). 2021 May 26;10(6):852. doi: 10.3390/antiox10060852 (PMC8229501; doi:10.3390/antiox10060852)
Supplement: Supplementary file 1 [file antioxidants-10-00852-s001.zip › antioxidants-1238092-supplementary.pdf]

Supplementary 1. Peak areas of metabolites representing their concentration or relative levels in extracts used to develop heatmap plot.

No.	Metabolites	BR seed (Peak area)	BR sprout (Peak area)	RC seed (Peak area)	RC sprout (Peak area)
1.	L-Histidine	0.00	510000.00	0.00	530000.00
2.	L-Asparagine	130000.00	120000.00	54000.00	67000.00
3.	Ornithine	63000.00	110000.00	0.00	140000.00
4.	γ-Aminobutryic acid	0.00	150000.00	0.00	180000.00
5.	Adenine	0.00	0.00	0.00	100000.00
6.	Quinic acid	0.00	49000.00	0.00	2100.00
7.	DL-o-Tyrosine	0.00	230000.00	0.00	230000.00
8.	Uridine	0.00	550000.00	0.00	460000.00
9.	L-Phenylalanine	72000.00	470000.00	0.00	500000.00
10.	trans-Cinnamic acid	0.00	70000.00	0.00	78000.00
11.	L-Tryptophan	0.00	250000.00	0.00	250000.00
12.	Actinonin	380000.00	340000.00	380000.00	210000.00
13.	m-Coumaric acid	0.00	0.00	0.00	70000.00
14.	Genipin	0.00	55000.00	0.00	330000.00
15.	Sinapic acid	110000.00	530000.00	75000.00	600000.00
16.	Glycitin	0.00	170000.00	0.00	310000.00
17.	3-Furoic acid	180000.00	130000.00	170000.00	0.00
18.	Leucine	0.00	150000.00	0.00	150000.00
19.	D-Serine	29000.00	73000.00	0.00	69000.00
20.	pyroglutamic acid	160000.00	320000.00	100000.00	280000.00
21.	L-Arginine	110000.00	180000.00	52000.00	130000.00
22.	Sinigrin	1200000	0.00	5300000.00	0.00
23.	+)-Epicatechin	0.00	0.00	68000.00	0.00
24.	Lysine	0.00	190000.00	0.00	250000.00

The values are presented as peak area representing the levels of metabolites in the extracts. Zero (0.00) peak area indicates that the corresponding metabolite was not detected in the respective extract.
